# A Review of the Piezoelectric Electromechanical Impedance Based Structural Health Monitoring Technique for Engineering Structures

**DOI:** 10.3390/s18051307

**Published:** 2018-04-24

**Authors:** Wongi S. Na, Jongdae Baek

**Affiliations:** Department of Infrastructure Safety Research, Korea Institute of Civil Engineering and Building Technology, Gyeonggi-Do 10223, Korea; jdbaek@kict.re.kr

**Keywords:** structural health monitoring, piezoelectric transducers, electromechanical impedance, non-destructive testing, impedance-based health monitoring

## Abstract

The birth of smart materials such as piezoelectric (PZT) transducers has aided in revolutionizing the field of structural health monitoring (SHM) based on non-destructive testing (NDT) methods. While a relatively new NDT method known as the electromechanical (EMI) technique has been investigated for more than two decades, there are still various problems that must be solved before it is applied to real structures. The technique, which has a significant potential to contribute to the creation of one of the most effective SHM systems, involves the use of a single PZT for exciting and sensing of the host structure. In this paper, studies applied for the past decade related to the EMI technique have been reviewed to understand its trend. In addition, new concepts and ideas proposed by various authors are also surveyed, and the paper concludes with a discussion of the potential directions for future works.

## 1. Introduction

The birth of smart materials such as piezoelectric (PZT) transducers has aided in revolutionizing the field of structural health monitoring (SHM) based on non-destructive testing (NDT) methods. One of the relatively new NDT methods, known as the electromechanical impedance (EMI) technique, has been a focus of international research for two decades; however, it has not been fully commercialized due to many existing limitations. In general, SHM is usually applied on-site, where the majority of inspection work is visual [1]. For this reason, inspectors require knowledge of the possible damage locations prior to inspection. Furthermore, inspecting a large scale civil infrastructure can be dangerous and time consuming, leading to an inefficient SHM system. In addition, critical part inspections of structures usually require NDT methods that involve large and heavy equipment, increasing the difficulty when monitoring structures such as cable-stayed and long-span suspension bridges.

A well-designed SHM system can minimize the overall maintenance costs of a structure by detecting damage at an early age, allowing action to be taken early to prevent further damage. One possible approach is the EMI technique, as it uses a single PZT transducer that acts as both actuator and sensor [2]. The advantage of the technique is that it has the ability to detect internal damage at a relatively low cost. In addition, since it uses high frequency range excitation, vibrations caused from the outside environment including vehicles and wind will not have a significant influence on the EMI technique. However, most of the research has been applied under a well-controlled environment, or inside a laboratory. In addition, issues such as durability and repeatability performance of the EMI technique are being continuously investigated.

Excellent reviews of the EMI technique can be found in previous studies, including Park et al. [3], Yang et al. [4] and Annamdas and Soh [5]. For this reason, our review of the EMI technique in this study is mainly focused on the investigations performed during the past decade, to give readers an understanding of how the technique has been improved thus far. The first two parts of the study focus on the theory and how to perform the EMI technique in order to give a quick overview of the EMI technique. The next part reviews applications and studies related to frequency ranges of the EMI technique, and how several authors proposed a method of creating a suitable frequency range if there are none to select from. In addition, various damage identification studies using artificial neural network algorithms are reviewed, and practical issues of the EMI technique are discussed. The finite element modeling (FEM) simulation work applied by researchers is also discussed. The last part of this study deals with future issues including EMI technique such as bond durability, PZT deterioration, limited sensing range, reference signature issues and the possibility of incorporating the use of a drone into the EMI technique for an efficient SHM system. 

## 2. Theory behind the EMI Technique

The theoretical development of the EMI technique was first introduced by Liang et al. [2] and has been studied by numerous researchers ever since. This technique utilizes a single PZT transducer, usually with a size of 10 mm^2^, to excite the host structure above 20 kHz, at which point the response is acquired using the same PZT transducer. The PZT is typically attached to the surface of the host structure using an adhesive, and a low voltage of 1V is used to deliver excitation to the structure. The 1-D model introduced by Liang et al. [2] is shown in Equation (1), which confirms that the electrical admittance Y(ω) (inverse of impedance) of the PZT is directly related to the mechanical impedance of the structure $Z_{s}\left(\omega \right)$. Thus, any changes in the properties of the structure can be identified by monitoring the changes in the electrical impedance of the attached PZT transducer. The remaining variables of ω, a, $\varepsilon_{33}^{T}$, δ, $d_{3x}^{2}$, ${\bar{Y}}_{xx}^{E}$ represent the input frequency, geometric constant, dielectric constant, loss tangent, coupling constant and Young’s modulus, respectively. Since the dielectric constant $\varepsilon_{33}^{T}$ is temperature-sensitive, affecting the imaginary part of the impedance only, ideally the real part of the impedance signature should be used for the EMI technique, as variations in the signature can cause false alarms [3,6].

(1) $$Y\left(\omega \right)=i\omega a\left(\varepsilon_{33}^{T}\left(1-i\delta \right)-\frac{Z_{s}\left(\omega \right)}{Z_{s}\left(\omega \right)+Z_{a}\left(\omega \right)}d_{3x}^{2}{\bar{Y}}_{xx}^{E}\right)$$

Various authors including Sun et al. [7], Zhou et al. [8], Esteban [9] and Park et al. [10] have also pioneered theoretical work regarding the EMI method during that time, where Xu and Liu [11] was the first to consider the adhesive layer regarding the impedance model where it was represented as 1-D spring-mass-damper system, placing in series with the structure. Regarding 2-D impedance models, Zagrai and Giurgiutiu [12] derived a theoretical model for a circular 2-D structure and validated with the results from experiments. The model considers axial and flexural vibrations of a target structure taking into account for both structural and sensor dynamics. The proposed model (Equation (2)) showed promising results compared to the experimental data where $\varphi_{a}=\omega r_{a}/c$. Here, $r_{a}$ is the radius of a disk, $k_{p}^{2}$ is the planar coupling, χ(ω) is the dynamic stiffness ratio, ν is Poisson’s ratio, $J_{0}$ and $J_{1}$ are the Bessel functions of first kind, order zero and one, respectively.

(2) $$Z\left(\omega \right)={\left\{i\omega C\left(1-k_{p}^{2}\right)\times \left[1+\frac{k_{p}^{2}}{1-k_{p}^{2}}\frac{\left(1+\nu \right)J_{1}\left(\varphi_{a}\right)}{\varphi_{a}J_{0}\left(\varphi \right)-\left(1-\nu \right)J_{1}\left(\varphi_{a}\right)-\chi \left(\omega \right)\left(1+\nu \right)J_{1}\left(\varphi_{a}\right)}\right]\right\}}^{-1}$$

**Figure 1:**
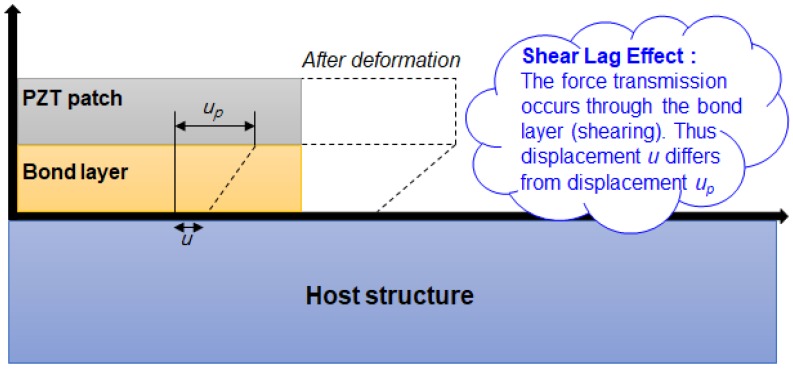
Bond layer and piezoelectric (PZT) transducer deformation [14].

Bhalla et al. [13] proposed a simplified 2-D impedance models considering shear lag effect caused by the adhesive bond layer between the PZT transducer and the host structure (Figure 1). The derived model shown in Equation (3) was compared with a previous work of Bhalla and Soh [14] showing the shear lag phenomenon reasonably well. Here, l and h are the dimensions of the PZT, $\bar{T}$ is the complex tangent ratio theoretically equal to tan(κl)/κl where κ is the wave number, and $Z_{a,eff}$ and $Z_{s,eff}$ are the effective impedance of the PZT and the host structure, respectively [14].

(3) $$\bar{Y}=G+Bj=4\omega j\frac{l^{2}}{h}\left[\bar{\varepsilon_{33}^{T}}-\frac{2d_{31}^{2}\bar{Y^{E}}}{\left(1-\nu \right)}+\frac{2d_{31}^{2}\bar{Y^{E}}}{\left(1-\nu \right)}\left(\frac{Z_{a,eff}}{Z_{s,eff,eq}+Z_{a,eff}}\right)\bar{T}\right]$$

Realizing the limitations (such as PZT shape and size) of the 1-D and 2-D models developed by various authors, Annamdas and Soh [15,16] presented a new 3-D model utilizing 3D actuations and PZT transducer. The model was validated with experiments of embedded and surface bonded transducer specimens. However, these single PZT-structure interaction models presented another issue when modeling multiple PZT-structure scenarios as previous models neglected the mass of the PZT transducers. Thus, Annamdas and Soh [17] developed a multiple PZT-structure (MPZT-S) model to consider the mass influence of multiple PZT transducers. The proposed model for predicting impedance by the authors is shown in Equation (4). Here, *N* is the number of PZT transducers with the dimension *L*, *W* and *H*. $Y_{R}=\bar{Y}\left(1-\nu \right)/\left(1+\nu \right)\left(1-2\nu \right)$, R=ν/(1−ν) and $\lambda_{1}$, $\lambda_{2}$, $\lambda_{3}$ are the response factors along directions *X*, *Y* and Z, respectively. Lastly, the equations for $A_{0}$, $C_{0}$ and $E_{0}$ can be found in the aforementioned literature.

(4) $$\begin{array}{c} {\bar{Y}}^{A}=N^{2}\frac{j\omega LW}{2H}[\bar{\varepsilon_{33}}+Y_{R}\{d_{31}\lambda_{1}\{[NA_{0}\sin kL-d_{31}]+R[NC_{0}sin\;kW-d_{32}]+\\ R[E_{0}k\cos k2H-d_{33}]\}+d_{32}\lambda_{2}\{R[NA_{0}\sin kL-d_{31}]+[NC_{0}sin\;kW-d_{32}]+\\ R[E_{0}k\cos k2H-d_{33}]\}+d_{33}\lambda_{3}\{R[NA_{0}\sin kL-d_{31}]+R[NC_{0}sin\;kW-d_{32}]+\\ \left[E_{0}k\cos k2H-d_{33}]\}\}\right] \end{array}$$

## 3. Applying the EMI Technique

### 3.1. Impedance Measuring Hardware

**Figure 2:**
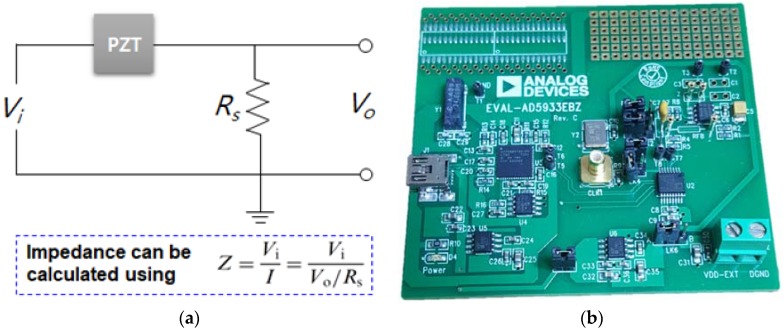
Impedance measuring device: (**a**) circuit for approximating impedance [18]; (**b**) photo of AD5933 evaluation board.

Since the introduction of the EMI technique, it has generally been applied using an impedance analyzer such as the Agilent 4194 A. However, the high cost of such devices created a demand for lower-cost ways of applying the EMI technique, as an impedance analyzer can cost up to US$40,000. Peairs et al. [18] introduced a low-cost method for applying the EMI technique using an FFT analyzer with the simple circuit (voltage divider) shown in Figure 2a. Using this approach, the impedance (*Z*) can be approximated by dividing the input voltage (*V_i_*) by the current through the sensing resistor (*I*). The development of a low-cost EMI technique has continued to be investigated by various researchers. Xu and Giurgiutiu [19] used a function generator HP33120A with a two-channel DAQ card, and more work on the low-cost system can be found in the work of Baptista and Vieira Filho. [20], Bhalla et al. [21] and Panigrahi et al. [22].

The EMI technique is a local damage detection method that is very effective for assessing the health of a nearby area. However, it is a method that can be difficult to manage when monitoring large structures, as it may involve hundreds to thousands of piezoelectric transducers. As the traditional wired systems with a large number of sensors can be very costly, this has highlighted the significance of wireless sensor nodes. The benefits of the wireless systems are well summarized by Spencer et al. [23] and Lynch and Loh [24]. Thus far, various authors have researched wireless systems [25,26] with the AD5933 impedance measurement chip manufactured by Analog Devices, Inc. At the size of a small coin, the chip itself is equipped with FFT functionality, an analog to digital converter and digital to analog converter. In addition, the company also commercialized a small impedance measuring device using the chip known as the ‘AD5933 Evaluation Board’ shown in Figure 2b, which can measure the impedance up to 100 kHz. The board is currently sold for less than US$100, and many researchers have investigated the EMI technique using this device.

One of the advantages of the EMI technique is that a single PZT transducer acts as both an actuator and sensor, simultaneously. In general, PZT ceramics have been used for majority of research related to the EMI technique where piezoelectric diaphragms (buzzers) have also proven to be effective for conducting the technique [27,28,29]. However, due to the ceramic nature of the conventional PZT ceramics, the brittle property makes them vulnerable to breakage and the difficulty in attaching it onto curved surfaces cannot be ignored. For this reason, various authors [30,31,32,33,34,35] have investigated using macro-fiber composite (MFC) created from NASA Langley Research Center [36]. MFC transducer consists of multiple layers of adhesive, interdigitated electrodes and polyimide film where piezoelectric materials are sandwiched between. This allows it to be both flexible and durable, making it suitable to be attached onto curved surfaces such as pipe structures. Thus, one can expect to see more research using MFC transducers for EMI technique in the future.

### 3.2. Statistical Metrics for Damage Quantification

When the properties of a structure change (e.g., due to damage, variations in temperature, etc.), the impedance signature also changes, with greater variations subjected to larger damage. For this reason, once the impedance signatures are acquired, it is necessary to quantify the severity of damage. In general, there are 4 different statistical equations used for the quantitative assessment of the signatures [37]. These are root mean square deviation (RMSD), mean absolute percentage deviation (MAPD), covariance (Cov) and correlation coefficient (CC), and are represented in Equations (5)–(8). Here, *Re*${\left(Z_{k}\right)}_{i}$ represents the reference impedance signature (real part) and *Re*${\left(Z_{k}\right)}_{j}$ the corresponding signature (real part). *N* is the number of impedance signatures, with the symbols $\bar{Z}$ signifying mean values and $\sigma_{Z}$ signifying standard deviation.

(5) $$RMSD={\left(\sum_{k=1}^{N}{\left[Re{\left(Z_{k}\right)}_{j}-Re{\left(Z_{k}\right)}_{i}\right]}^{2}/\sum_{k=1}^{N}{\left[Re{\left(Z_{k}\right)}_{i}\right]}^{2}\right)}^{1/2}$$

(6) $$MAPD=\frac{1}{N}\sum_{k=1}^{N}\left|\left[Re{\left(Z_{k}\right)}_{j}-Re{\left(Z_{k}\right)}_{i}\right]/Re{\left(Z_{k}\right)}_{i}\right|$$

(7) $$Cov=\frac{1}{N}\sum_{k=1}^{N}\left[Re{\left(Z_{k}\right)}_{j}-Re{\left(\bar{Z}\right)}_{j}\right]\cdot \left[Re{\left(Z_{k}\right)}_{i}-Re{\left(\bar{Z}\right)}_{i}\right]$$

(8) $$CC=\frac{1}{N\sigma_{Z_{j}}\sigma_{Z_{i}}}\sum_{k=1}^{N}\left[Re{\left(Z_{k}\right)}_{j}-Re{\left(\bar{Z}\right)}_{j}\right]\cdot \left[Re{\left(Z_{k}\right)}_{i}-Re{\left(\bar{Z}\right)}_{i}\right]$$

Thus far, most of the researchers who have been investigating the EMI technique have used one of these 4 statistical equations, with the *RMSD* metric having been used the most. Tseng and Naidu [38] investigated the performance of the 4 different statistical metrics, and it was found through experiments that *RMSD* and *MAPD* were more suitable for locating and characterizing the growth of damage, whereas covariance and *CC* were more suitable for identifying the increase in damage size at a fixed location. Tawie and Lee [39] used the EMI technique to monitor concrete curing and strength gain for up to 28 days using the three statistical metrics (*RMSD*, *MAPD* and *CC*). The PZT transducer was attached to the 150 mm^3^ sized concrete specimen where impedance was measured on days 7, 14 and 28. From the study, the authors discovered that *MAPD* correlated better than both the *RMSD* and *CC* metrics. Xu and Jiang [40] investigated the EMI technique by monitoring bolt loosening of a concrete-steel composite girder using *RMSD*, *MAPD* and *CCD*. A number of PZT transducers were surface-bonded to the upper flange of the steel girder and the concrete slab, where bolt loosening was identified by monitoring impedance signatures. Hu et al. [41] focused on detecting damage on a concrete slab (500 mm × 300 mm × 50 mm) using *RMSD*, *CCD* (*CCD* = 1 − *CC*) and a new damage index proposed by the authors, Ry/Rx. The results showed that the frequency range below 100 kHz performed better than ranges above this frequency range for damage identification, with the authors confirming that the proposed new damage index showed better results. Wandowski et al. [42] used Chessboard Distance (*CB*), a statistical metric other than the aforementioned four equations, to evaluate the performance of the EMI technique on composite structures subjected to damage. Here, the authors found that *CB* performed better than *RMSD* as *CB* values increased, with an increased level of damage at different temperatures.

Other statistical metrics for analyzing the impedance signatures include average square deviation (ASD), united mechanical impedance (UMI), ellipse damage index (EDI) and others, and new metrics in the field of the EMI technique are being proposed by various researchers [43,44,45]. However, the use of a single type of metric for EMI based damage detection has some limitations, as small damage at close range to the PZT transducer can present the same results as larger damage further away. One approach to overcoming this problem could be to combine several statistical metrics to increase the potential of correctly identifying and locating structural damage. 

## 4. Investigations on the EMI Technique

### 4.1. Applications

The real application of the EMI technique was adopted for the first time by Annamdas and Yang (2012) in Singapore, Telok Blangah district [46]. The work presented monitoring results of the soil excavation conducted for construction of new mass road transport station using the EMI technique. PZT transducers were installed on a temporary support structure (for preventing soil collapse during construction) where impedance signatures were monitored up to a year. Although no significant damage was reported in the structure, it was found that the EMI technique was effective detecting changes in loading caused from the surrounding soil. 

Although that there are still many issues to be dealt with regarding the EMI technique, one can expect to see more applications of the EMI technique onto real structures in the future. Since the EMI technique can detect any changes in structural property, the range of its application is very wide. The research for this technique has reached beyond mechanical and civil engineering fields as various authors have attempted the EMI technique to be used for biological applications. Tabrizi et al. [47] investigated using the EMI technique for assessing the stability of dental implants after surgical placement. The experiment involved chemically degrading the prepared specimens with nitric acid where calcium loss amount was correlated with the impedance signature. Regardless of the promising results obtained from the experiments, the authors stated that the approach should be tested for repeatability to bring a step closer to be accepted in the field of dental implantology. Bhalla and Suresh examined the possibility of using the EMI technique for monitoring conditions of human and rabbit bones [48]. The study included detecting cracks and fracture as well as the healing process after a fracture. In addition, changes in bone density was also monitored using the conductance of the signatures.

Regarding mechanical and civil engineering field applications, the EMI technique has proven to be effective in various areas including crack and load damage of various structures, debonding of composite adhesive layer, corrosion of metallic structures, bolt loosening of bridge components, prestressed concrete (PSC) related force loss and concrete strength predictions. Detecting a crack or notch using the EMI technique of structures can be said to be one of the most investigated areas of the EMI technique. This includes detecting damage subjected to different structure types such as metal, composites and concrete structures [49,50,51,52,53,54,55,56,57,58,59]. A good correlation relationship can be seen between the distance and severity of damage compared to a statistical metric. In general, closer and severer damage will results in a larger statistical metric value where such results can be seen in some of the recent studies. Corrosion and bolt loosening detection of metallic structures have also been continuously investigated over the past several years. Regarding composite materials, since adhesive bonding is a preferred choice over the mechanical fastening approach, adhesive layer monitoring has been an important field in EMI technique. Some of the latest research includes composite-to-composite and composite-to-concrete debonding detections [60,61,62,63,64,65]. Kim et al. [66] investigated on the possibility of utilizing the EMI technique for predicting the prestress forces loss for PSC girder bridges. In this work, impedance signatures were measured at 5 prestress levels where the frequency was measured up to 1000 kHz. From the results, signature variations were clearly visible subjected to different prestress levels. Other studies related to concrete have also been active during the past several years as many authors applied the EMI technique for predicting concrete strength. Lu et al. [67] proposed an idea of using ‘Smart Probe’ to monitor the strength development of cementitious materials. Dynamic modulus of elasticity of cementitious materials was predicted with the proposed analytical model where it showed good correlation with the compressive strength of the mortar. Talakokula et al. [68] focused on monitoring the hydration process of cementitious materials for the first 28 days. The study measured both the real and imaginary part of admittance (inverse of impedance) where the results showed that the magnitude of the signatures gradually decreased with time. The authors conclude by stating that the approach proposed in the work has the potential to be applied to in situ hydration monitoring of reinforced concrete structures. More research in the field of concrete strength prediction can be found in recent studies [69,70,71,72,73,74,75]. 

### 4.2. Selecting or Creating a Suitable Frequency Range

Although that the frequency domain analysis of the EMI technique has been researched in vast amounts, very little has been published related to time domain analysis methods where it uses the excitation circuit proposed by Baptista and Vieira [20]. From various authors, it has been known that time domain analysis methods are more sensitive for detecting damage compared to the conventional frequency domain analysis of the EMI technique [76,77,78]. However, it was found that the efficiency lied within detecting existence of damage and not on its exact location. For this reason, the remainder of the study will continue to focus of the frequency domain analysis of the EMI technique. The frequency range of the EMI technique is selected by manually seeking multiple peaks and setting that specific range for analysis. In previous studies, the frequency ranges used for the EMI technique have been up to 1000 kHz, with higher frequency ranges resulting in a smaller sensing region and lower frequency ranges resulting in a larger sensing region. In general, a frequency range below 400 kHz is ideal, with ranges below 80 kHz covering a relatively large area. Regarding frequency range selection, Yang and Divsholi [79] applied the EMI technique to a concrete mass in the frequency range from 30 kHz to 400 kHz. This range was divided into 6 sub-frequency groups (30–99.9 kHz, 100–149.9 kHz, 150–199.9 kHz, 200–249.9 kHz, 250–299.9 kHz, and 300–400 kHz) to evaluate the performance of each sub-frequency range against damage. The results of the experiment showed that damage close to the PZT changed the impedance signatures significantly in the higher frequency ranges, whereas damage far away from the PZT changed the signatures significantly in the lower frequency ranges. Min et al. [80] investigated the autonomous selection of a suitable frequency range for the EMI technique using an artificial neural network technique. The proposed artificial neural network (ANN) scheme allowed one to automatically identify a damage-sensitive frequency region to be used for the experiment applied in the study. 

**Figure 3:**
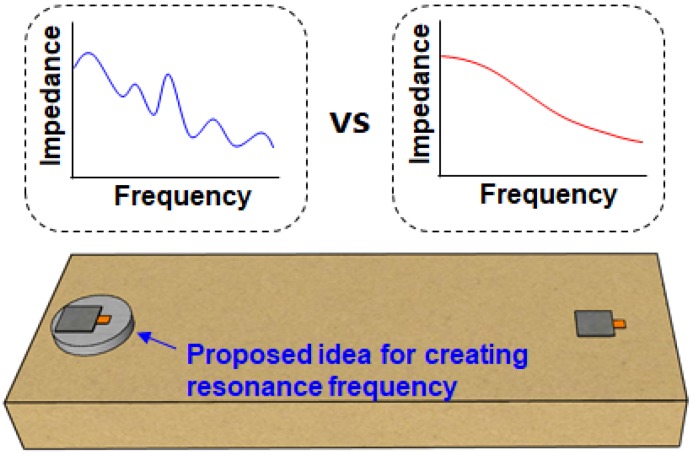
Resonance frequency creating concept [81].

If no peaks can be found when sweeping the frequency ranges to search a region with multiple peaks, the EMI technique may fail to detect any structural damage. Without any resonance, the changes in the impedance signature can be quite small. To solve this problem, Na and Lee proposed a method of creating a resonance frequency range when applying the EMI technique (Na and Lee [81]. The idea was to sandwich a metal disc between the PZT and the structure, where the resonance frequency of the metal disc is maintained regardless of the properties of the structure (Figure 3). Thus, the resonance frequency range could be changed simply by changing the size of the metal disc. This idea was extended by using a steel wire to apply the EMI technique [82], which allowed the EMI technique to be applied to any kind of surface, as the tip of the wire could be attached virtually anywhere. The resonance frequency ranges could be changed by using different sized PZT transducers for the steel wire EMI technique. Thus, when the EMI technique is applied and no resonance frequencies with peaks are found, an alternative solution is to create a suitable frequency range with multiple peaks for successful damage detection. 

### 4.3. Artificial Neural Networks and EMI Technique

**Figure 4:**
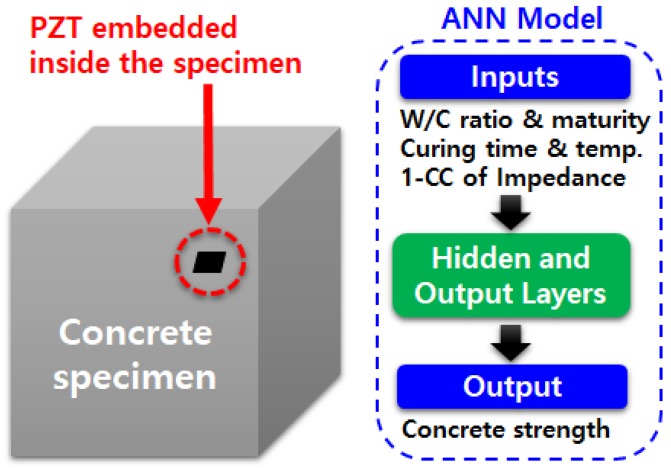
Concept of concrete strength prediction with artificial neural network (ANN) [87].

The ultimate goal of SHM technique is to identify the location of damage and its size, and estimate the remaining service life of the target structure. Using the EMI technique, many authors have studied how the impedance signatures change when subjected to different sizes and locations of damage. One well known technique of locating damage is by using an artificial neural network (ANN), and various authors have already evaluated how an ANN can perform when incorporated into the EMI technique. When an ANN is applied to the EMI technique, a decent amount of training is usually required for the algorithm to successfully identify damage. Some common types of ANN techniques can be found in Giurgiutiu and Kropas-Hughes [83], in which the researchers have applied the EMI technique to aluminum alloy based circular plates of 100 mm diameter and 1.6 mm thickness. The probabilistic neural network (PNN) algorithm was applied with a featured extraction algorithm for the aforementioned study. Min et al. applied a multi-layer feed-forward NN with a back-propagation algorithm in conjunction with the EMI technique for damage identification [84]. A series of experiments was carried out to evaluate the proposed technique subjected to loose bolts and crack damages on both lab scale pipe structure and on a box girder bridge. Na and Lee [85] used a glass-epoxy composite plate with a size of 200 mm × 200 m × 3.5 mm with 3 PZT transducers attached to each of the 3 corners of the plate, with the purpose of the study being to predict the location of damage using the developed PNN algorithm. In this study, 6 different areas were classified for testing, and 24 damaged holes out of 30 were correctly predicted, an 80% accuracy rate. The study concludes with the statement that increasing the number of training data could improve the outcome of the results. Palomino et al. [86] focused on the classification of two different types of damages (cracks and rivet losses) on an aluminum panel using PNN and fuzzy cluster analysis methods. The authors discovered that the location of the PZT transducer is vital for successful damage identification. Oh et al. [87] combined ANN with the EMI technique for predicting the strength of concrete by monitoring the impedance signatures for 28 days during the curing of the concrete. For training of the ANN algorithm, water-and-cement ratio, curing time, curing temperature, maturity and CC value calculated from the impedance signatures were used (Figure 4). The proposed ANN model estimated the strength of prepared concrete specimens with negligible error. de Oliveira et al. [88] proposed an enhanced strategy for damage detection via Savitzky–Golay (SG) filter, PNN and simplified fuzzy ARTMAP network, with these three methods applied to an experiment using an aluminum plate containing three PZT transducers. The results showed a promising outcome in terms of successfully detecting damage.

The trend of combining the ANN algorithm with the EMI technique can be said to be increasing, as the statistical metrics such as RMSD, MAPD, covariance and CC are not sufficient to quantify, locate and identify what kind of damage the structure has received.

### 4.4. Practical Issues

**Figure 5:**
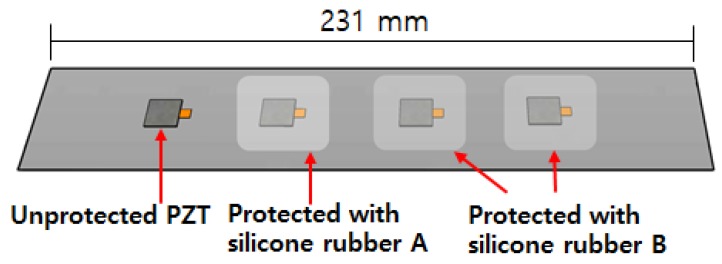
Test specimen setup testing PZT durability (c.f. Yang et al., 2008 [4]).

Since the introduction of the EMI technique, countless studies have been carried out in this area, with many reporting promising results. However, most of the studies performed were under laboratory conditions or mainly theoretically based, which raises questions regarding their potential in real-life applications, particularly under harsh environments. Although structure damage changes the impedance signature, other factors such as variations in temperature and the durability of PZT transducers can also cause the signatures to change as well. Yang et al. [4] investigated the signature repeatability of PZT transducers both indoor and outdoor up to 15 months, and found that silicone rubber protection over the PZT resulted in RMSD values that were lower than unprotected PZTs (Figure 5). In addition, the authors experimentally evaluated how the bonding layers of the PZT transducers and variations in the temperature influenced the impedance signatures. Sun et al. [6] applied the EMI technique under a temperature varying condition and discovered that a rise in temperature seemed to soften the overall stiffness of the host structure, causing the resonance frequency range to shift and the peak amplitudes to change. The authors also used cross-correlation to minimize the signature variation due to temperature change by horizontally shifting the signature. Park et al. [89] discovered that the real part of the impedance signatures should be preferred to the imaginary part, as they found out that the real part signature of the free PZT patch changed only negligibly with temperature variations. Furthermore, the authors suggested another technique to compensate the signature variations due to temperature changes; many other researchers have also studied this area. Grisso and Inman [90] proposed a technique for separating the temperature variation effect from sensor defects, in which a frame structure was used to conduct experiments under various temperatures. The results of their experiments reveal that the measured susceptance slope showed a linear relationship to temperature variations. Sepehry et al. [91] combined ANN based on radial basis function (RBF) with the EMI technique to compensate the temperature effect. A steel plate and gas pipe were used for the study, and damage was artificially created by drilling a hole and loosening bolt, respectively. Wandowski et al. [92] used carbon fibre reinforced polymer samples to evaluate the proposed temperature compensation algorithm. The principle of the 2-step algorithm is to first shift the signature in the horizontal direction using cross-correlation and then in the vertical direction using signal normalization with its root mean square values. 

Although multiple techniques have been proposed to compensate the temperature effect of the EMI technique, it is extremely difficult to compensate this effect completely. Sometimes the variations in impedance signatures can be unpredictable, as some peaks may shift or increase/decrease in amplitude more than others [93].

### 4.5. Experiment vs. Simulation

**Figure 6:**
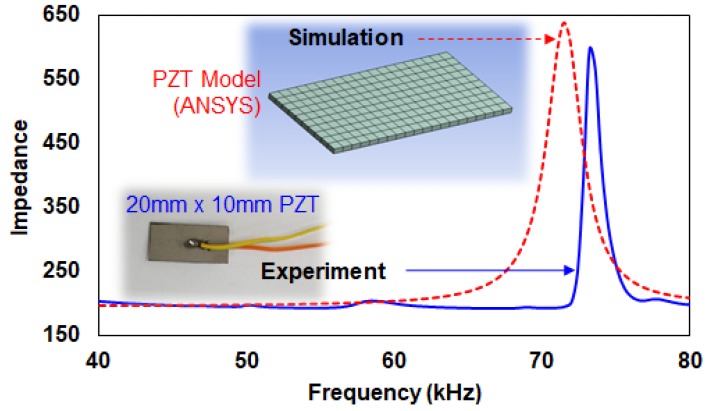
Simulation vs. experiment impedance signatures on a 20 mm × 10 mm PZT.

To gain a better understanding of an engineering system, modeling is an essential field of research, and a large number of studies are published in this area each year. Considering the EMI technique, PZT-structure interaction models have been intensively developed. However, there are limitations to these solutions when they are applied to real structures, as they can be only applied to simple structures such as beams, shells and plates with simple boundary conditions. As such, finite element method (FEM) became known as an alternative solution. With powerful FEM tools, the efficiency of FE modeling is increasing every day, particularly with the advancement of computer technology. Figure 6 shows two impedance signatures for a 20 mm × 10 mm × 0.5 mm PZT transducer where one was measured using AD5933 evaluation board and the other using a FEM simulation tool. The resonance frequency ranges for both impedance signatures are within 65–80 kHz range.

A well summarized work on FE-based simulation of PZT-structure interaction can be found in the PhD dissertation by Lalande [94], in which the author used ANSYS software to show promising outcomes on ring and shell structures. Tseng and Wang [95] applied both experimental and numerical studies on 2 concrete beam structures, which led to the result of the RMSD values increasing when crack damage increased and when crack damage occurred closer to the PZT transducers. Lim and Soh [49] investigated the possibility of using the EMI technique for estimating the remaining fatigue life of a 1-D aluminum structure using ANSYS 12.1, incorporating the linear elastic fracture mechanics theory. In their work, the authors stated that the CCDM (cross correlation deviation mean) results showed better outcomes compared to the RMSD results when characterizing crack length. Yang et al. [96] used ANSYS to apply the EMI technique on various models including bonding layers on an aluminum beam, and the results showed excellent agreement compared to the experimental data up to 1000 kHz. In addition, the authors simulated the EMI technique from 30 °C to 60°C in the frequency range up to 400 kHz, also showing good agreement with the experimental results. Hamzeloo et al. [97] used the EMI technique to perform both experiment and simulation (using ABAQUS) on aluminum and steel specimens. The test specimens were hollow cylinders with different thicknesses, and the test was performed in the frequency range from 10 to 40 kHz. Using the RMSD metric, the results showed that accumulation of damage does not always increase the RMSD values. Rugina et al. [98] reported on the use of the EMI technique on thin circular plates, comparing the results of the analytical method, FE simulation and experiments. At undamaged state, all the impedance signatures showed good agreement when compared to one another in the frequency range up to 40 kHz. Djemana et al. [99] focused on detecting and locating damage on an aluminum beam structure with the application of an extreme learning machine-based algorithm developed for the study. An overall accuracy of 84.5% was achieved in terms of predicting the location of a single damage in structure with the aid of FEM simulations.

Thus far, the majority of the FEM simulation studies related to the EMI technique have used either ANSYS or ABAQUS. Since the EMI technique is a non-modeled method that is heavily reliant on acquired impedance signatures, incorporating FEM may enable the achievement of the ultimate goal of locating, identifying and quantifying the size of damage.

## 5. Future Work

### 5.1. Bond Durability, PZT Deterioration, Sensing Range and Reference Signature

For the EMI technique to be applied to SHM system, the durability of the PZT transducer and the bonding layer must be high. While many PZT manufacturers claim that the PZT deterioration rate is less than 5% per decade, environmental factors must be taken into account. Environmental factors such as changes in temperature over a long period of time can lead to thermal fatigue, while rain or sea water exposure could result in acidic and alkaline attacks. In addition, the PZT transducer should endure both the mechanical and electrical cyclic loading. Although there are ways to protect the PZT transducers, such as embedding them into the structure or covering them up with silicon or epoxy, the PZTs cannot be replaced if they are damaged. In addition, changes in the properties of the cover (silicon or epoxy) can cause the impedance signatures to change, causing a fault alarm. For this reason, the influence of these factors needs to be eliminated through compensation algorithms, or perhaps by using a control or multiple PZT transducers.

Regarding sensing range, the EMI technique is a local method that uses high frequency excitation. For this reason, the sensing range is limited to several meters, depending on the properties of the PZT and the host structure. As such, cost issues arise when dealing with large structures, as a vast number of PZT transducers need to be installed. This shows that using the EMI technique alone to develop SHM system is very difficult, and a global method (e.g., accelerometer on bridge cables) should be incorporated to create an efficient SHM system. A well-known combination of the EMI technique with the guided wave-based technique has been investigated in Giurgiutiu et al. [100] where it was found that the EMI technique was found to be suitable for damage detection in the near field while the guided wave based technique was more suitable for far-field damage detection [101,102,103,104,105]. Lastly, one of the first steps for conducting the EMI technique is to acquirea reference signature in a ‘no damage’ state. This reference signature is then compared to a damaged state at a later stage to identify any damage to the structure. As it is virtually impossible to acquire a ‘no damage’ state impedance signature for existing structures, future studies could be focused on using the EMI technique without the use of a reference signature, either by using the method alone or in combination with other NDT techniques.

### 5.2. Drone EMI Technique

As the Fourth Industrial Revolution approaches, various areas of technology have become key items of interest around the globe. Technologies such as artificial intelligence (AI) and drones can be said to be closely related to the EMI technique. Although the amount of ANN work in conjunction to the EMI technique is increasing every day, the combination of drones with the EMI technique (or any other non-visual based NDT methods) is in the very beginning stage of research. Although visual inspection-based drone monitoring technology is advancing, this technology has limitations when searching for internal damage or even small cracks, which can be difficult to identify using a camera. Some of the work on visual based SHM using drones can be found in Ellenberg et al. [106], Ham et al. [107], Flammini et al. [108] and Reagan et al. [109]. On the other hand, the EMI technique is a contact-based method which has the potential of detecting internal damage of structures. 

**Figure 7:**
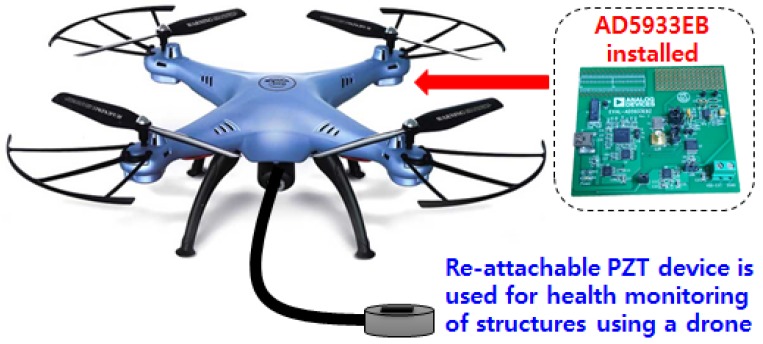
Drone electromechanical (EMI) technique concept [110].

The first work on combining the EMI technique with drone was performed by Na and Baek [110], using a re-attachable PZT transducer using a magnet (Figure 7). In this research, the authors discovered that the impedance signature did not change during the hovering state, regardless of the drone vibrations. However, re-attaching the PZT transducer caused the impedance signature to change where additional future work is required to overcome to problem. Regardless of the signature change due to the re-attachment, the authors found that the proposed drone-EMI based technique showed potential for detecting thickness reduction in steel structures.

### 5.3. Multi-functional Sensing Possibilities

By 2050, it has been predicted that up to 70% of the world’s population will live in urban areas and with this fact, the importance of developing a smart-city is becoming more and more important. One of the vital aspects for achieving an efficient smart-city lies within the field of sensors. With a vast number of sensors installed around the city, management of the overall system may depend on factors such as the number of sensors. For this reason, the importance of multi-functional sensors will become more important as this may reduce the total number of sensors required for an effective smart city. As mentioned in a previous section, the impedance signatures during the EMI technique has been experimentally proven to vary with change in temperatures. This gives the possibility of the EMI technique not only to detect damage but also predict the temperature of the host structure, simultaneously. With this idea, it may be possible to reduce two sensors into a single sensor to reduce the overall number of sensors required for a structural health monitoring system.

**Figure 8:**
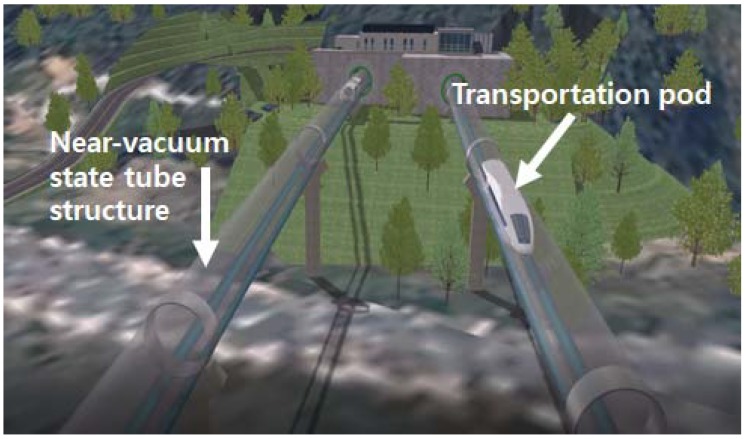
Hyperloop concept from Korea Institute of Civil Engineering and Building Technology (KICT).

With sensing range limitations of the EMI technique, one may suggest that the real application of such technique can be very limited. However, a future technology such as the ‘Hyperloop’ system can demand such technique as the EMI technique is effective for detecting damage at a very early stage. The Hyperloop system is a new type of ground transportation technology which can theoretically travel up to 1200 km per hour, moving at a speed faster than an airplane. The concept of the technology can be seen in Figure 8 where the basic idea is that a pod travels inside a near-vacuum stated tube structure to reduce air resistance. The two well-known USA companies, Hyperloop Transportation Technologies (HTT) and Virgin Hyperloop One are currently researching the technology to make it into a reality where details can be found in [111]. Such advanced technology will require sensors to detect damage at a very early age for an efficient maintenance system. Furthermore, the tube length could be built up to several hundreds to thousands of kilometers, where a large number of sensors will be possibly required. For this reason, multi-functional sensors could be an important factor to achieve such technology. 

## 6. Conclusions

In this work, we have reviewed studies that have been conducted on the application of EMI technique, focusing on the latest research in the field. While the EMI technique has been researched for over two decades, there are still various problems to be solved in its application. These problems include the limited sensing range, the need to select a suitable frequency range, the need to compensate for environmental factors such as temperature variations, efficient statistical metrics etc., which need to be solved to achieve one of the ultimate goals of locating and identifying damage type. For this reason, one of the trends is that the studies using ANN has increased everyday where it has proven to give promising outcome for the EMI technique. As well, ANN has been investigated not only for measuring structural damage but for a variety of applications including selecting suitable frequency range, temperature compensation, predicting concrete strength, bolt loosening etc. 

The EMI technique has great potential, with numerous studies being conducted to solve various problems. However, since most of the studies have been conducted under lab conditions, future research should be more focused on real structures in order to identify critical problems using the EMI technique. Nevertheless, continuous research by experts and advancement in computer technologies will bring new solutions and ideas, with FEM simulations becoming more effective for bringing the EMI technique one step closer to commercialization.
